# Correlates of Positivity Among a Sample of Lebanese University Students

**DOI:** 10.3389/fpsyg.2022.880437

**Published:** 2022-04-25

**Authors:** Sara Moussa, Diana Malaeb, Sahar Obeid, Souheil Hallit

**Affiliations:** ^1^Faculty of Medicine, University of Balamand, Koura, Lebanon; ^2^College of Pharmacy, Gulf Medical University, Ajman, United Arab Emirates; ^3^Department of Social and Education Sciences, School of Arts and Sciences, Lebanese American University, Jbeil, Lebanon; ^4^School of Medicine and Medical Sciences, Holy Spirit University of Kaslik, Jounieh, Lebanon; ^5^Department of Psychology, College of Humanities, Effat University, Jeddah, Saudi Arabia; ^6^Department of Research, Psychiatric Hospital of the Cross, Jal Eddib, Lebanon

**Keywords:** positivity, flourishing, religious coping, experiences in life, financial burden, Lebanon

## Abstract

**Background:**

People say it is hard to stay truly positive in Lebanon. Studies showed that 63% of Lebanese young adults are highly dissatisfied with their country. In fact, young adults are the most vulnerable population to stressors in Lebanon since their future is at stake and it is their time to shape their lives in a country that cripples them. This study aimed to assess factors (flourishing, religious coping, experiences in life, and the economic burden) associated with positivity among a sample of Lebanese university students despite the various stressors they are facing on top of the economic collapse and the COVID-19 pandemic.

**Methods:**

This cross-sectional study was conducted between November and December 2021. A total of 333 participants (219 females and 114 males; mean age = 22.95 ± 4.79 years) was recruited through convenience sampling and snowball technique through several areas in Lebanon’s governorates. A linear regression taking the positivity score as the dependent variable was adopted and all variables that showed a correlation > │0.24│ in absolute value were entered in the final model as independent.

**Results:**

A linear regression taking the positivity score as the dependent variable showed that more positive experiences in life (Beta = 0.49; 95% CI 0.35–0.62), more flourishing (Beta = 0.10; 95% CI 0.05–0.14), living in rural area compared to urban (Beta = 3.06; 95% CI 2.02–4.11), and female gender (Beta = 1.56; 95% CI 0.50–2.61) were significantly associated with more positivity (Nagelkerke *R*^2^ of the model = 45.8%).

**Conclusion:**

This study demonstrated that the youth’s positivity is strongly affected by age, gender, residency, and the country they live in that will both directly and indirectly shape their life experiences and their ability to flourish and prosper. Along with all the efforts done to help during this collapse and alleviate the stress that young adults are enduring, follow-up studies are still needed to determine accurate coping techniques that pushes these young adults to think positively in a country where negativity reigns and all else fails.

## Background

People say it is hard to stay truly positive in Lebanon. This country has been under continuous conflict for more than four decades, putting millions of Lebanese at high risk for post-traumatic stress disorder (PTSD) and creating a complex concept known as collective trauma (El Hajj, 2021). On top of that, this small country has been recently paralyzed by many tragedies including the economic collapse, COVID-19, and the fourth of August Beirut port explosion, the world’s most powerful non-nuclear explosion (Farran, 2021). All these stressors that Lebanese young adults are subjected to have destroyed their lives and made them persons not being able to live up to their expectations anymore (Sfeir et al., 2020; Hussein, 2021). Given that positivity is a factor influenced by external and environmental factors, it qualifies as an important variable to explore during these hard times in Lebanon, especially in young adult students.

Emotion is an intriguing topic that has been widely studied and investigated. It is an important concept for every aspect of life and is at the core of the human experience (Plutchik, 1980). Traditionally, emotions have been categorized dichotomously, as negative or positive, unpleasant, or pleasant. However, studies have progressively shown that they are rather simultaneous (Russel, 1980). McGrath defines positive mindset as an overall attitude that is reflected in thinking, behavior, feeling, and speaking (McGrath, 2004). In fact, positivity empowers us to grow, seize new opportunities, and move on in life. It is both a skill and a mindset granted on someone (Gordon, 2019). If used correctly, this skill allows one to perceive the stress as less threatening and be able to cope with it effectively (Naseem and Khalid, 2010). According to a study done by the Michigan State University (2017), a person has about 12,000–60,000 thoughts per day. Of those, 80% are negative (Michigan State University, 2017). For example, negative events in childhood are significant risk factors for developing a pessimistic view of life and having pertinent negative thoughts (Read and Bentall, 2012). However, even though negative experiences are labeled as obstacles; those are inevitable and necessary for lifelong learning. On the other hand, positive life events were linked to higher self-esteem, self-acceptance, and positive perceptions of life (Brown and McGill, 1989). The States-of-Mind (SOM) model proves that a healthy balance of both positive and negative thoughts is essential for psychological wellbeing (Schwartz and Caramoni, 1989). Thus, reaching this balance is crucial nowadays to face the daily stressors that shape one’s life experiences, by partly alleviating strong anxiety and panic reactions, and providing a solid background for reflexive and rational behavior in these difficult times (Trzebiński et al., 2020).

Another factor strongly related to positivity is religiosity especially in strongly religious countries. A meta-analysis of longitudinal studies demonstrated that participation in public religious activities, for example, was significantly related to mental health, which was reflected by a better life satisfaction, wellbeing, and perception of life (Garssen et al., 2021). In fact, evidence supports the correlation between religion and lower risks of depression, anxiety, suicide ideations, stress-related symptoms, and specific mental health illnesses such as bipolarity (Oman and Lukoff, 2018). At the same time, higher levels of religious commitment were also strongly linked with increased dispositional gratitude, purpose in life, and post-traumatic growth (Sharma et al., 2017). The literature showed that these associations can be applied to all religions as long as they have these positive virtues. Forgiveness and altruism in religion are two main pathways that lead to a healthy positive mindset (Sharma and Singh, 2019). To put it differently, two studies conducted within the Islamic tradition (Francis et al., 2008; Tiliouine, 2014), four studies conducted within the Jewish tradition (Francis and Katz, 2002; Francis et al., 2004, 2017; Levin, 2012), and six studies conducted within the Christian tradition (Robbins and Francis, 1996; Francis and Katz, 2002; Francis et al., 2003, 2004, 2017; Ritter et al., 2014) led to the same conclusion that religious people are happier people regardless of their religion (Francis et al., 2017). However, regardless of one’s religion, the coping mechanisms can be either positive or negative. Positive religious coping strategies include seeking God’s love and care, reframing difficult situations as opportunities for growth, and turning to God in times of hardships to find strength and relief (O’Brien et al., 2019), whereas negative religious coping, often mistaken for religious or spiritual struggles, reflects a constant state of conflict within oneself with God, or with other people (Exline, 2013). Results of a study supported the hypotheses that different forms of religious coping are correlated with different psychological adjustments to stress (Ano and Vasconcelles, 2005). Another study additionally emphasized these findings by confirming that positive and negative religious coping were associated with higher and lower levels of wellbeing, respectively (Pargament et al., 2001).

In a study done on 988 Lebanese citizens, findings showed that 63% of Lebanese young adults are highly dissatisfied with their country, which cripples their flourishing in life (Bou-Hamad et al., 2021). Indeed, the relationship between flourishing and positivity is bidirectional. Fredrickson and Losada found that those who reported high positivity ratios were classified as flourishing; this manifested in their sense of fulfillment and ability to bounce back effectively from stressful situations (Gloria and Steinhardt, 2013). Diehl et al. explored the association between flourishing and positivity in depth in young adults. They concluded that flourishing adults lead meaningful, happy, and productive lives, whereas languishing adults, although free of clinically relevant mental illness, describe their lives as empty and stagnant (Diehl et al., 2011). This was also true when studied in young adults with cancer. They showed higher levels of positivity which allowed better internal integration and ultimately created a harmonious and balanced existence with their environment and outside world (Cho and Docherty, 2020).

The situation in Lebanon further worsened after the events of the last two years. A new Lebanese study showed that fear from the COVID-19 pandemic alongside the complex financial situation were associated with high levels of stress and anxiety among young adults (El Othman et al., 2021). Research has shown positive associations between depression and various indicators of financial stress such as debt or debt stress, financial hardship, or difficulties (Bridges and Disney, 2010). A 2022 systematic review that included 40 studies conducted on young adults, concluded that there is a positive association between financial stress and depression in both high-income and low- and middle-income countries but is generally stronger among populations with low income or wealth during such difficult times (Guan et al., 2022). Financial challenges are an important component of positivity, especially in young adults. Young adulthood is normally a time of growth, opportunity, and achieving important developmental milestones. They are more likely to have initiated their career, own a home, be married or in long-term partnerships, have children, and have more extensive social support networks (Salsman et al., 2019). All these stressors add to their economic burden in a time that already crippled their financial abilities to achieve these milestones, which will strongly affect their positivity.

On one hand, young adults are the most vulnerable population to the stressors in Lebanon since their future is at stake and it is their time to shape their lives in a country that cripples them. On another hand, little to no research has investigated the relationship between positivity and religious coping. In fact, only one study had been conducted on Lebanese young adults prior to the economic crisis, COVID-19 pandemic, and the Beirut Blast. This study explains how this population has always been underrepresented even in times of the civil war from 1975 until 1991, the continuous fighting for 16 years with the massacres and kidnappings (Abdel-Khalek, 2015). These circumstances molded the Lebanese life and personality, which was proved in the study with high levels of mental problems (Abdel-Khalek, 2015; Yaacoub et al., 2019). Therefore, the present study is unique in using an underrepresented sample in the literature who endured traumatic events in his childhood. Up until today, studies tackling these variables have either neglected one of them, did not target the young population, or have been conducted in a different time frame, which is a crucial element in this study. Therefore, this study aimed to assess factors (flourishing, religious coping, experiences in life, and the financial burden) associated with positivity among a sample of Lebanese university students despite the various stressors they are facing on top of the economic collapse and the COVID-19 pandemic. A secondary objective aimed to assess the mediating effect of religious coping in the relationship between experiences in life and positivity. We hypothesize that more flourishing, positive experiences in life and positive religious coping would be associated with more positivity, whereas more financial burden, negative experiences in life, and negative religious coping would be associated with less positivity.

## Materials and Methods

### Participants

This cross-sectional study was conducted between November and December 2021. A total of 333 participants (219 females and 114 males; mean age = 22.95 ± 4.79 years) were recruited through convenience sampling through several areas in Lebanon’s governorates. Participants received an online link to the survey through social networks. They were encouraged to visit the link, which would guide them to the consent form, purpose of the study, anonymity, and the questionnaire. There were no fees for participating in the study. Eligible participants to fill out the questionnaire were those aged between 18 and 30, who are enrolled in a university, regardless of the major, and who have given consent to respond willingly. Those who are outside this age range, are no longer university students, and those who refused to complete the survey were excluded.

### Minimal Sample Size Calculation

Using the G-power software (Faul et al., 2007), a minimum of 316 students was deemed necessary to have enough statistical power, based on a 5% risk of error, 80% power, *f* ^2^ = 2.5% and 10 factors to be entered in the multivariable analysis.

### Questionnaire

The questionnaire used was anonymous and in Arabic, the native language in Lebanon; it required approximately 10–15 min to complete. A sworn translator performed a forward translation, from English into Arabic and two healthcare professionals performed the back translation into English, for all scales. The two English versions were then compared to check for discrepancies, which were resolved by consensus. The questionnaire consisted of two parts. The first one assessed sociodemographic characteristics of satisfaction, and the number of persons in the household and the number of rooms in the house, excluding the bathroom and the kitchen, to calculate the household crowding index (the number of rooms divided by the number of persons; Melki et al., 2004). Regarding their financial burden, respondents were asked to answer the question “How much pressure do you feel with regard to your personal financial situation in general?” on a scale from 1 to 10, with 10 referring to overwhelming pressure.

The second part consisted of the following scales.

### Positivity Scale

Caprara et al. developed this scale *t* based on five studies to measure positivity, defined as the tendency to view life and experiences with a positive outlook (Caprara et al., 2012). Items in this scale such as “I am satisfied with my life” are scored on a basic numeric scoring from 1 (strongly disagree) to 5 (strongly agree). The total score, calculated by summing the eight items, ranged from 0 to 40, with higher scores indicating higher levels of positivity (Caprara et al., 2012). The Italian items were translated and then back translated into Spanish and English by two different bilingual experts who were fluent, respectively, in both Spanish and Italian, and Spanish and English. The English items were translated and then back translated by two different bilingual experts, fluent in both Japanese and English (Caprara et al., 2012; Cronbach’s alpha in the current study = 0.79).

### Flourishing Scale

This scale is a brief eight-item summary measure of the respondent’s self-perceived success in important areas such as relationships, self-esteem, purpose, and optimism (Diener et al., 2010). For example, “I lead a purposeful and meaningful life” tackles self-esteem, optimism, and purpose in life. It was developed by Diener et al. after evaluating a sample of 689 college students from six different locations. Each item is scored from 1 (strongly disagree) to 7 (strongly agree); thus, the possible range of final scores is from 8 (lowest possible) to 56 (highest possible). A high score represents a person with many psychological resources and strengths (Diener et al., 2010; Cronbach’s alpha in the current study = 0.95).

### The Brief Religious Coping Methods Scale (Brief RCOPE Scale)

This scale was developed by Professor Kenneth Pargament, who is known for his interest in religion-centered studies. In fact, this scale is only one of the many scales that he developed related to this topic. The scale was developed strictly in English. Nearly all of the studies that used the Brief RCOPE have been conducted in the United States and Western Europe and did not need translation. In one notable exception, Khan and Watson (2006) translated it into Urdu in their study of Muslim Pakistani university students. In our case, permission to use and translate this scale to Arabic was obtained from the author, Professor Kenneth Pargament. The scale consists of seven positive coping items and seven negative coping items. Positive items were generated from seven different subscales from the original RCOPE and the seven negative items originate from five different subscales (Pargament et al., 2011). Positive items exist as such “sought God’s love and care,” whereas negative items exist as such “questioned God’s love for me.” As for the scoring, each item’s score ranges from 1 (not at all) to 4 (a great deal). Then, the positive items are summed to create a positive Brief RCOPE score ranging from 7 to 24, and the negative items are also summed to create a negative Brief RCOPE score in a similar manner. However, a total Brief RCOPE score is not doable because the scales are not generally highly correlated with each other (Pargament et al., 2011; Cronbach’s alpha values in the current study were 0.90 for the positive religious coping and 0.94 for the negative religious coping).

### Scale of Positive and Negative Experience

This is the second scale developed by Diener et al. after the Flourishing Scale discussed above. This scale compromises 12 items. It has the advantage of assessing a broad range of experiences instead of a precise one, within a certain time frame (last 4 weeks). For instance, it assesses “how much one has felt positive during the past 4 weeks” from 1 (very rarely or never) to 5 (very often or always). It does that for six positive feelings and six negative feelings. By doing that, it produces a score for positive feelings, a score for negative experiences, each ranging from 6 to 30. Then, the two scores can be combined to create a balance score in which the negative feelings score is subtracted from the positive feelings score, and the resultant difference score can vary from −24 (unhappiest possible) to 24 (highest affect balance possible). A respondent with a very high score of 24 reports that she or he rarely or never experiences any of the negative feelings, and very often or always has all the positive feelings (Diener et al., 2010; Cronbach’s alpha values in the current study were 0.88 for the positive subscale and 0.84 for the negative subscale).

### Statistical Analysis

There was no missing data in both databases since all questions were required in the online forms. SPSS software version 23 was used to conduct data analysis. Cronbach’s alpha values were recorded for reliability analysis of all scales and subscales. Weighting to the general population was applied according to gender and education. The positivity score was normally distributed, with its skewness and kurtosis varying between −2 and + 2 (George, 2011). The Student *t* was used to compare two means, whereas the Pearson’s correlation test was used to compare two continuous variables. A forward linear regression was conducted to check for correlates associated with positivity.

The PROCESS SPSS Macro version 3.4 model four (Hayes, 2017) was used to estimate the indirect effects of each positive/negative religious coping in the relationship between positive/negative experiences in life and positivity. Three pathways were computed as: Pathway A determined the regression coefficient for the effect of positive/negative experiences in life on positive/negative religious coping, Pathway B examined the association between positive/negative religious coping and positivity, and Pathway C′ estimated the direct effect of positive/negative experiences in life and positivity. Pathway AB calculated the indirect intervention effects; a significant mediation was determined if the macro generated bias-corrected bootstrapped 95% confidence intervals of the indirect effect did not include zero (Hayes, 2017). All variables that showed a correlation > │0.24│ in absolute value were entered in the multivariable and mediation models as independent ones to have a parsimonious model (Vandekerckhove et al., 2014). Significance was set at a *p* < 0.05.

## Results

### Sociodemographic and Other Characteristics of the Participants

A total of 333 students participated in this study; their mean age was 22.95 ± 4.79 years, with 65.8% females. Other characteristics are summarized in Table 1.

**Table 1:** Sociodemographic and other characteristics of the participants (*N* = 333).

Variable	N (%)
**Sex**	
Male	114 (34.2%)
Female	219 (65.8%)
**Marital status**	
Single	266 (80.1%)
Married	66 (19.9%)
**Region of living**	
Urban	210 (63.1%)
Rural	123 (36.9%)
**Education**	
Secondary or less	18 (5.4%)
University	315 (94.6%)
	**Mean ± SD**
**Age (in years)**	22.95 ± 4.79
**Household crowding index**	1.09 ± 0.55
**Financial burden**	5.68 ± 2.66

### Bivariate Analysis

The bivariate analysis results are shown in Tables 2 and 3. A higher mean positivity score was seen in those living in urban cities compared to rural ones. Higher household crowding index, more financial burden, more negative religious coping, and more negative experiences in life were significantly associated with less positivity, whereas more flourishing, positive religious coping, and positive experiences in life were significantly associated with more positivity.

**Table 2:** Bivariate analysis of the categorical variables associated with positivity.

Variable	Positivity		
	Mean ± SD	*p*	Effect size
**Sex**			
Male	26.63 ± 6.78	0.052	0.25
Female	25.28 ± 3.81		
**Marital status**			
Single	25.57 ± 5.27	0.203	0.19
Married	26.46 ± 4.06		
**Region of living**			
Urban	24.59 ± 5.18	**<0.001**	0.87
Rural	28.44 ± 3.52		
**Education**			
Secondary or less	25.62 ± 5.04	0.372	0.13
University	26.26 ± 5.13		

Numbers in bold indicate significant values of *p*.

**Table 3:** Correlations of the continuous variables with positivity.

Variable	Positivity	FS	PRC	NRC	PEL	NEL	Age	HCI	FB
Positivity	1								
Flourishing score (FS)	0.52^***^	1							
Positive religious coping (PRC)	0.32^***^	0.42^***^	1						
Negative religious coping (NRC)	−0.26^***^	−0.25^***^	0.06	1					
Positive experiences in life (PEL)	0.59^***^	0.57^***^	0.54^***^	−0.30^***^	1				
Negative experiences in life (NEL)	−0.23^***^	0.06	0.30^***^	0.36^***^	−0.02	1			
Age	0.06	0.04	0.44^***^	0.21^***^	0.14^**^	0.26^***^	1		
Household crowding index (HCI)	−0.22^***^	0.05	0.04	0.25^***^	−0.15^**^	−0.16^**^	0.27^***^	1	
Financial burden (FB)	−0.16^**^	−0.37^***^	−0.21^***^	0.32^***^	−0.14^*^	−0.07	−0.14^*^	−0.03	1

Numbers in bold indicate significant values of *p*; numbers in the table refer to Pearson’s correlation coefficients.

* *p < 0.05*;

** *p < 0.01*;

*** *p < 0.001*.

### Multivariable Analysis

A linear regression taking the positivity score as the dependent variable showed that more positive experiences in life, more flourishing, living in rural area compared to urban and female gender were significantly associated with more positivity (Table 4).

**Table 4:** Multivariable analysis: Linear regression (using the ENTER model) taking the positivity score as the dependent variable.

	Beta	β	*p*	95% CI
Gender (females vs. males^*^)	1.56	0.15	**0.004**	0.50–2.61
Religion of living (rural vs. urban^*^)	3.06	0.28	**<0.001**	2.02–4.11
Flourishing score	0.10	0.22	**<0.001**	0.05–0.14
Positive religious coping	0.01	0.01	0.880	−0.08–0.09
Negative religious coping	−0.04	−0.05	0.310	−0.12–0.04
Positive experiences in life	0.49	0.42	**<0.001**	0.35–0.62

* Reference group.

Nagelkerke *R*^2^ = 45.8%; Beta, unstandardized beta; *β*, standardized beta; CI, confidence interval; and numbers in bold indicate significant values of *p*.

## Mediation Analysis

The results showed that positive/negative religious coping did not have an indirect effect in the association of positive/negative experiences in life and positivity, after adjustment over the following variables: gender, region of living, flourishing score, and the other experience in life variable (Table 5).

**Table 5:** Mediation analysis: Direct and indirect effects of the associations between positive/negative religious coping in the relationship between positive/negative experiences in life and positivity.

		Direct effect			Indirect effect	
Mediator						
	Effect	SE	*p*	Effect	SE	95% BCa
**Model 1: Positive experiences in life as the independent variable**						
Positive religious coping	0.20	0.05	<0.001	0.01	0.01	−0.01–0.03
Negative religious coping	0.20	0.05	<0.001	0.001	0.01	−0.01–0.01
**Model 2: Negative experiences in life as the independent variable**						
Positive religious coping	−0.28	0.04	<0.001	0.004	0.01	−0.01–0.02
Negative religious coping	−0.28	0.04	<0.001	0.001	0.01	−0.01–0.01

*Results were adjusted over gender, region of living, flourishing score, and the other experience in life variable.*

## Discussion

This study demonstrated that more positive experiences in life, more flourishing, living in a rural area compared to urban, and female gender were significantly associated with more positivity.

In this study, we found that higher positive experiences in life were associated with more positivity, whereas higher negative experiences were associated with less positivity, corroborating the findings of previous studies (Diener et al., 2000; Miller et al., 2008; Seta et al., 2008; O’Brien and Ellsworth, 2012; Catalino et al., 2014). Individuals with more frequent experiences of positive emotions were more likely to prioritize positivity in their daily lives (Catalino et al., 2014). This was further proved in countries that valued their citizens’ wellbeing by ensuring a healthy environment to live in compared to countries where chaos reigns (Diener et al., 2000). In fact, citizens that faced more stressors and negative experiences in life were less likely to display positivity (Diener et al., 2000). The psychological sequelae of exposure to negative experiences have shed important light on the mental health effects that could accompany living through the hardships of life (Miller et al., 2008). In fact, the relationship between life experiences and positivity can be felt just by observing people around you. People naturally feel more positive when they are exposed to a highly positive event (Seta et al., 2008). Thus, negative experiences may produce different effects than positive experiences do (O’Brien and Ellsworth, 2012).

We found that flourishing in life is significantly associated with more positivity in agreement with previous papers (Fredrickson and Losada, 2005; Reschly et al., 2008; Scorsolini-Comin et al., 2013; Asebedo and Seay, 2015; Prizmić-Larsen et al., 2020). A previous study showed that people who thrive to live a full and prosperous tend to have a better perception of life and more positive thoughts as evidenced in their life planning and decisions (Asebedo and Seay, 2015). Furthermore, previous results showed that the mean ratio of positive to negative emotions was high for individuals classified as flourishing and below threshold for those not flourishing (Fredrickson and Losada, 2005). It has also been reported that this association can be bidirectional. Authentic happiness and positivity are a great motive to further seek life satisfaction and wellbeing, which aspires one to flourish and blossom (Scorsolini-Comin et al., 2013). To put things into perspective, flourishing can be evidenced by more engagement in school, for example. Students who have greater positive thoughts had higher levels of engagement and better adaptive coping (Reschly et al., 2008). Therefore, flourishing and leading a purposeful life helps in focusing on the positive life events and thoughts while making it easier to process negativity rather than overlooking it (Prizmić-Larsen et al., 2020).

We found that residents of rural areas reported higher positivity than those living in urban settings. In spite of the various studies done to investigate the urban–rural difference in positivity, the relationship is still complex. Some scholars suggest that urban people have better mental health than do rural people (Li and Li, 2014), whereas others suggest the opposite (Robins et al., 1981; Blazer et al., 1985; Kovess-Masféty et al., 2005; Fogelholm et al., 2006; Srivastava, 2009; Reichert et al., 2020). Mikael et al. believed that rural old people had better mental health than urban old people (Fogelholm et al., 2006). Despite the many benefits it offers, the rapid worldwide urbanization we are facing predisposes us to a higher risk of common psychiatric disorders (Reichert et al., 2020). An Indian study showed that India’s urbanization affected mental health through the influence of various factors such as the overcrowded environment, high levels of stressors, and reduced social support (Srivastava, 2009). The milestone US Epidemiologic Catchment Area Study showed the same. Individuals living in urban settings had a significantly higher risk of major depression (2.4%; Robins et al., 1981) than did those living in rural areas (1.1%; Blazer et al., 1985). Moreover, most European studies have shown fewer positive emotions in urban areas, compared with rural areas. This effect was mostly prominent in the Netherlands and in the United Kingdom (Kovess-Masféty et al., 2005). Consequently, the prevalence of positivity is slightly but significantly higher in residents of rural areas compared to urban areas, possibly due to differing population characteristics.

In addition, female gender was significantly associated with higher levels of positivity. These results are contradictory to most previous findings (Crocker and Graham, 1995; Roothman et al., 2003; Calvete and Cardenoso, 2005; Pascual et al., 2012), but consistent with others (Halberstadt et al., 1988; Zweig, 2015). On one hand, females often tend to have lower levels of positivity compared to males. For example, a study showed that female adolescents have lower levels of positive thinking and higher scores of negative problem orientation (Calvete and Cardenoso, 2005). Another study was done on a multicultural availability sample of 378; men scored higher on positive automatic thoughts, constructive thinking, and cognitive flexibility (Roothman et al., 2003). This was also proven when tested on 762 adolescents. Results obtained indicate that boys tend to experience positive emotions more frequently than girls, while girls tend to experience negative emotions more frequently than boys (Pascual et al., 2012). These results were replicable on athletes as well, where females used higher levels of seeking social support for emotional reasons and increasing effort to manage goal frustration while males experienced higher levels of positive affect (Crocker and Graham, 1995). On the other hand, other studies demonstrated the opposite. College students’ conversations about their emotional experiences were analyzed for smiling frequency and duration, with females having smiled more than males, which reflects their higher level of positivity (Halberstadt et al., 1988). In fact, the female–male positivity gap has been a topic of interest to Zweig, who examined it in 73 different countries. This paper provided evidence that when comparing men and women with the same life circumstances, women are happier and more positive than men in nearly a quarter of the countries (Zweig, 2015). Thus, these gender differences in positivity levels are an intriguing topic to dig deeper into.

Our results showed that positive/negative religious coping did not have an indirect effect in the association of positive/negative experiences in life and positivity. To our knowledge, no previous studies have investigated the mediating role of religious coping in this association. In Lebanon, one can hypothesize that a person’s positive and negative life experiences directly affect his positivity levels, independently of his religious coping methods. Thus, based on our findings and the literature, we are sure that both religiosity and life experiences have a direct effect on positivity but the indirect correspondence of religiosity in the link between positivity and life experiences seems to be unclear. Therefore, this can be an open door for future research to dig deeper into the mediating role of religious coping in the association of life experiences in life and positivity.

## Implications of the Study

Our study adds to the narrow body of research revolving around the relationship between positivity and its different correlates in individuals aged between 18 and 28. Our results might constitute a starting point to researchers to evaluate in-depth the association of positivity with any of the variables mentioned, whether in Lebanon or in another country. Understanding these results would help developing additional strategies that will be implemented by faculty members, giving more value to the correlates mentioned that have a major effect on the lifestyle of university students in Lebanon. In fact, it might even give the people in charge in the universities the motivation to observe these variables more carefully among their young adult students. Furthermore, understanding the stressors that young adults are subject to in Lebanon would help establish more preventative guidelines and mental health awareness campaigns. Identifying the correlates of positivity can also be translated into improved interventions. Finally, our findings would let the students know that mental health issues are very common and can be dealt with very easily, if accepted. There should be no social stigma attached.

## Limitations

As all studies, our cross-sectional study has limitations; the data’s nature limits the ability to draw causal conclusions. Due to the COVID-19 pandemic, a self-administered questionnaire was distributed *via* an online form. This can pose a risk for information bias considering the risk of misunderstanding the questions by either under or over-estimating them by the participants. Information bias can also be because positivity is a subjective variable that relies on the person’s opinion. The chosen sample’s nature (convenient sampling *via* the snowball technique) also prompts a risk of selection bias aggravated by refusal rates, which does not allow the generalization of the results. Thus, further studies with larger samples are required to better assess the associations found in this study. The scales are not validated in Lebanon; thus, results should be interpreted with caution. Residual confounding bias is also possible since not all factors associated with positivity were considered in this study. However, this study can be useful as a preliminary template to further develop future studies.

## Conclusion

To our knowledge, no previous research has looked into a direct link between positivity and these correlate in Lebanon especially in these times. Further studies are needed in order to better understand the underlying mechanisms between this association and well as the factors that mediate the link between these variables. Thus, by shedding light on the findings of this research, we can assume that one’s positivity is strongly affected by his age, gender, residency, and the country he lives in that will both directly and indirectly shape his life experiences and his ability to flourish and prosper. Therefore, universities in Lebanon play a pivotal role and are encouraged to offer information aimed at increasing mental health awareness, to facilitate services for students at risk, and to be an alleviating factor instead of a stressing one, in the life of their students. Along with all the efforts done to help during these hard times in Lebanon and alleviate the stress that young adults are enduring, follow-up studies are still needed to determine accurate coping techniques that push these young adults to think positively in a country facing very difficult situations.

## Data Availability Statement

The original contributions presented in the study are included in the article/supplementary material, further inquiries can be directed to the corresponding author.

## Ethics Statement

The studies involving human participants were reviewed and approved by the Psychiatric Hospital of the Cross. The patients/ participants provided their written informed consent to participate in this study when submitting the online form.

## Author Contributions

SM and SH conceived and designed the survey. SH was involved in the statistical analysis and data interpretation. SM wrote the manuscript and involved in the data collection. DM, SH, and SO reviewed the manuscript. All authors contributed to the article and approved the submitted version.

## Conflict of Interest

The authors declare that the research was conducted in the absence of any commercial or financial relationships that could be construed as a potential conflict of interest.

## Publisher’s Note

All claims expressed in this article are solely those of the authors and do not necessarily represent those of their affiliated organizations, or those of the publisher, the editors and the reviewers. Any product that may be evaluated in this article, or claim that may be made by its manufacturer, is not guaranteed or endorsed by the publisher.

## References

[ref1] Abdel-Khalek A. M. (2015). Happiness, health, and religiosity among Lebanese young adults. Cogent Psychol. 2:1035927. doi: 10.1080/23311908.2015.1035927

[ref2] Ano G. G. Vasconcelles E. B. (2005). Religious coping and psychological adjustment to stress: a meta-analysis. J. Clin. Psychol. 61, 461–480. doi: 10.1002/jclp.20049, PMID: 15503316

[ref3] Asebedo S. D. Seay M. C. (2015). From functioning to flourishing: applying positive psychology to financial planning. J. Financ. Plan. 28, 50–58.

[ref4] Blazer D. George L. K. Landerman R. Pennybacker M. Melville M. L. Woodbury M.. (1985). Psychiatric disorders: a rural/urban comparison. Arch. Gen. Psychiatry 42, 651–656. doi: 10.1001/archpsyc.1985.01790300013002 4015306

[ref5] Bou-Hamad I. Hoteit R. Harajli D. (2021). Health worries, life satisfaction, and social well-being concerns during the COVID-19 pandemic: insights from Lebanon. PLoS One 16:e0254989. doi: 10.1371/journal.pone.0254989, PMID: 34324533 PMC8321151

[ref6] Bridges S. Disney R. (2010). Debt and depression. J. Health Econ. 29, 388–403. doi: 10.1016/j.jhealeco.2010.02.003 20338649

[ref7] Brown J. D. McGill K. L. (1989). The cost of good fortune: when positive life events produce negative health consequences. J. Pers. Soc. Psychol. 57, 1103–1110. doi: 10.1037/0022-3514.57.6.1103, PMID: 2614661

[ref8] Calvete E. Cardenoso O. (2005). Gender differences in cognitive vulnerability to depression and behavior problems in adolescents. J. Abnorm. Child Psychol. 33, 179–192. doi: 10.1007/s10802-005-1826-y, PMID: 15839496

[ref9] Caprara G. V. Alessandri G. Eisenberg N. Kupfer A. Steca P. Caprara M. G.. (2012). The positivity scale. Psychol. Assess. 24, 701–712. doi: 10.1037/a0026681 22250591

[ref10] Catalino L. I. Algoe S. B. Fredrickson B. L. (2014). Prioritizing positivity: An effective approach to pursuing happiness? Emotion 14, 1155–1161. doi: 10.1037/a0038029, PMID: 25401290 PMC5533095

[ref11] Cho E. Docherty S. L. (2020). Beyond resilience: A concept analysis of human flourishing in adolescents and young adults with cancer. ANS Adv. Nurs. Sci. 43, 172–189. doi: 10.1097/ANS.0000000000000292 31922987

[ref12] Crocker P. R. Graham T. R. (1995). Coping by competitive athletes with performance stress: gender differences and relationships with affect. Sport Psychol. 9, 325–338. doi: 10.1123/tsp.9.3.325

[ref13] Diehl M. Hay E. L. Berg K. M. (2011). The ratio between positive and negative affect and flourishing mental health across adulthood. Aging Ment. Health 15, 882–893. doi: 10.1080/13607863.2011.569488, PMID: 21562989 PMC3158962

[ref14] Diener E. Napa-Scollon C. K. Oishi S. Dzokoto V. Suh E. M. (2000). Positivity and the construction of life satisfaction judgments: global happiness is not the sum of its parts. J. Happiness Stud. 1, 159–176. doi: 10.1023/A:1010031813405

[ref15] Diener E. Wirtz D. Tov W. Kim-Prieto C., Choi, D.-w., Oishi S. Biswas-Diener R. (2010). New well-being measures: short scales to assess flourishing and positive and negative feelings. Soc. Indic. Res., 97, 143–156. doi: 10.1007/s11205-009-9493-y.

[ref16] El Hajj M. (2021). Prevalence and associated factors of post-traumatic stress disorder in Lebanon: a literature review. Asian J. Psychiatr. 63:102800. doi: 10.1016/j.ajp.2021.102800, PMID: 34340165

[ref17] El Othman R. Touma E. El Othman R. Haddad C. Hallit R. Obeid S.. (2021). COVID-19 pandemic and mental health in Lebanon: a cross-sectional study. Int. J. Psychiatry Clin. Pract. 25, 152–163. doi: 10.1080/13651501.2021.1879159 33587678

[ref18] Exline J. J. (2013). “Religious and spiritual struggles,” in APA Handbook of Psychology, Religion, and Spirituality: Vol. 1. Context, Theory, and Research. ed. Pargament K. I. (Washington, DC: American Psychological Association), 459–475.

[ref19] Farran N. (2021). Mental health in Lebanon: Tomorrow’s silent epidemic. Ment. Health Prev. 24:200218. doi: 10.1016/j.mhp.2021.200218, PMID: 34660191 PMC8503814

[ref20] Faul F. Erdfelder E. Lang A.-G. Buchner A. (2007). G* power 3: A flexible statistical power analysis program for the social, behavioral, and biomedical sciences. Behav. Res. Methods 39, 175–191. doi: 10.3758/BF03193146, PMID: 17695343

[ref21] Fogelholm M. Valve R. Absetz P. Heinonen H. Uutela A. Patja K.. (2006). Rural—urban differences in health and health behaviour: a baseline description of a community health-promotion programme for the elderly. Scand. J. Public Health 34, 632–640. doi: 10.1080/14034940600616039, PMID: 17132597

[ref22] Francis L. J. Katz Y. J. (2002). “Religiosity and happiness: a study among Israeli female undergraduates,” in Research in the Social Scientific Study of Religion, *Vol*. 13. Netherlands: Brill, 75–86.

[ref23] Francis L. J. Katz Y. J. Yablon Y. Robbins M. (2004). Religiosity, personality, and happiness: a study among Israeli male undergraduates. J. Happiness Stud. 5, 315–333. doi: 10.1023/B:JOHS.0000048460.35705.e8

[ref24] Francis L. J. Ok Ü. Robbins M. (2017). Religion and happiness: a study among university students in Turkey. J. Relig. Health 56, 1335–1347. doi: 10.1007/s10943-016-0189-8, PMID: 26832334

[ref25] Francis L. J. Robbins M. White A. (2003). Correlation between religion and happiness: a replication. Psychol. Rep. 92, 51–52. doi: 10.2466/pr0.2003.92.1.51, PMID: 12674256

[ref26] Francis L. J. Sahin A. Al-Failakawi F. (2008). Psychometric properties of two Islamic measures among young adults in Kuwait: the Sahin-Francis scale of attitude toward Islam and the Sahin index of Islamic moral values. J. Muslim Ment. Health 3, 9–24. doi: 10.1080/15564900802035201

[ref27] Fredrickson B. L. Losada M. F. (2005). Positive affect and the complex dynamics of human flourishing. Am. Psychol. 60, 678–686. doi: 10.1037/0003-066X.60.7.678, PMID: 16221001 PMC3126111

[ref28] Garssen B. Visser A. Pool G. (2021). Does spirituality or religion positively affect mental health? Meta-analysis of longitudinal studies. Int. J. Psychol. Relig. 31, 4–20. doi: 10.1080/10508619.2020.1729570

[ref29] George D. (2011). SPSS for Windows Step by Step: A Simple Study Guide and Reference, 17.0 Update, 10/e. Chennai, India: Pearson Education India.

[ref30] Gloria C. T. Steinhardt M. A. (2013). Flourishing, languishing, and depressed postdoctoral fellows: differences in stress, anxiety, and depressive symptoms. J. Postdoc. Aff. 3, 1–9.

[ref31] Gordon K. (2019). Toxic Positivity: Unintentional Gashlighting. DK Leadership. Available at: https://www.cityline.tv/wp-content/uploads/2019/11/Toxic-Positivity.pd (Accessed November 20, 2019).

[ref32] Guan N. Guariglia A. Moore P. Xu F. Al-Janabi H. (2022). Financial stress and depression in adults: A systematic review. PLoS One 17:e0264041. doi: 10.1371/journal.pone.0264041, PMID: 35192652 PMC8863240

[ref33] Halberstadt A. G. Hayes C. W. Pike K. M. (1988). Gender and gender role differences in smiling and communication consistency. Sex Roles 19, 589–604. doi: 10.1007/BF00289738

[ref34] Hayes A. F. (2017). Introduction to Mediation, Moderation, and Conditional Process Analysis: A Regression-Based Approach. New York: Guilford publications.

[ref35] Hussein D. (2021). The Onset of depression in young adults based on income group and the effect of the economic crisis in Lebanon.

[ref36] Khan Z. H. Watson P. J. (2006). Construction of the Pakistani religious coping practices scale: correlations with religious coping, religious orientation, and reactions to stress among Muslim university students. Int. J. Psychol. Relig. 16, 101–112. doi: 10.1207/s15327582ijpr1602_2

[ref37] Kovess-Masféty V. Alonso J. de Graaf R. Demyttenaere K. (2005). A European approach to rural—urban differences in mental health: the ESEMeD 2000 comparative study. Can. J. Psychiatry 50, 926–936. doi: 10.1177/070674370505001407, PMID: 16494262

[ref38] Levin J. (2012). Religion and positive well-being among Israeli and diaspora Jews: findings from the world values survey. Ment. Health Relig. Cult. 15, 709–720. doi: 10.1080/13674676.2011.617002

[ref39] Li J. Li C. (2014). Health difference of the elderly between the rural and urban districts. Popul. J. 36, 37–47.

[ref40] McGrath P. (2004). The burden of the ‘RA RA’positive: survivors’ and hospice patients’ reflections on maintaining a positive attitude to serious illness. Support. Care Cancer 12, 25–33. doi: 10.1007/s00520-003-0547-4, PMID: 14579137

[ref41] Melki I. Beydoun H. Khogali M. Tamim H. Yunis K. (2004). Household crowding index: a correlate of socioeconomic status and inter-pregnancy spacing in an urban setting. J. Epidemiol. Community Health 58, 476–480. doi: 10.1136/jech.2003.012690, PMID: 15143115 PMC1732777

[ref42] Michigan State University (2017). Challenge your negative thoughts. Available at: https://www.canr.msu.edu/news/challenge_your_negative_thoughts (Accessed March 5, 2017).

[ref43] Miller K. E. Omidian P. Rasmussen A. Yaqubi A. Daudzai H. (2008). Daily stressors, war experiences, and mental health in Afghanistan. Transcult. Psychiatry 45, 611–638. doi: 10.1177/1363461508100785, PMID: 19091728

[ref44] Naseem Z. Khalid R. (2010). Positive thinking in coping with stress and health outcomes: Literature Review. J. Res. Educ. Eff. 4, 43–49.

[ref45] O’Brien E. Ellsworth P. C. (2012). Saving the last for best: A positivity bias for end experiences. Psychol. Sci. 23, 163–165. doi: 10.1177/0956797611427408 22241815

[ref46] O’Brien B. Shrestha S. Stanley M. A. Pargament K. I. Cummings J. Kunik M. E.. (2019). Positive and negative religious coping as predictors of distress among minority older adults. Int. J. Geriatr. Psychiatry 34, 54–59. doi: 10.1002/gps.4983, PMID: 30375027

[ref47] Oman D. Lukoff D. (2018). Mental Health, Religion, and Spirituality. Why Religion and Spirituality Matter for Public Health. Berlin: Springer, 225–243.

[ref48] Pargament K. Feuille M. Burdzy D. (2011). The brief RCOPE: current psychometric status of a short measure of religious coping. Religions 2, 51–76. doi: 10.3390/rel2010051

[ref49] Pargament K. I. Tarakeshwar N. Ellison C. G. Wulff K. M. (2001). Religious coping among the religious: The relationships between religious coping and well-being in a national sample of Presbyterian clergy, elders, and members. J. Sci. Study Relig. 40, 497–513. doi: 10.1111/0021-8294.00073

[ref50] Pascual A. Etxebarria I. Ortega I. Ripalda A. (2012). Gender differences in adolescence in emotional variables relevant to eating disorders. Int. J. Psychol. Psychol. Ther. 12, 59–68.

[ref51] Plutchik R. (1980). Emotion. A Psychoevolutionary Synthesis. New York: Harper & Row.

[ref52] Prizmić-Larsen Z. Kaliterna-Lipovčan L. Larsen R. Brkljačić T. Brajša-Žganec A. (2020). The role of flourishing in relationship between positive and negative life events and affective well-being. Appl. Res. Qual. Life 15, 1413–1431. doi: 10.1007/s11482-019-09743-y

[ref53] Read J. Bentall R. P. (2012). Negative childhood experiences and mental health: theoretical, clinical and primary prevention implications. Br. J. Psychiatry 200, 89–91. doi: 10.1192/bjp.bp.111.096727, PMID: 22297585

[ref54] Reichert M. Braun U. Lautenbach S. Zipf A. Ebner-Priemer U. Tost H.. (2020). Studying the impact of built environments on human mental health in everyday life: methodological developments, state-of-the-art and technological frontiers. Curr. Opin. Psychol. 32, 158–164. doi: 10.1016/j.copsyc.2019.08.026, PMID: 31610407

[ref55] Reschly A. L. Huebner E. S. Appleton J. J. Antaramian S. (2008). Engagement as flourishing: The contribution of positive emotions and coping to adolescents’ engagement at school and with learning. Psychol. Sch. 45, 419–431. doi: 10.1002/pits.20306

[ref56] Ritter R. S. Preston J. L. Hernandez I. (2014). Happy tweets: Christians are happier, more socially connected, and less analytical than atheists on twitter. Soc. Psychol. Personal. Sci. 5, 243–249. doi: 10.1177/1948550613492345

[ref57] Robbins M. Francis L. J. (1996). “Are religious people happier. A Study among Undergraduates,” in Research in Religious Education. eds. Francis L. J. Kay W. K. Campbell W. S. (Leominster: Gracewing), 207–217.

[ref58] Robins L. N. Helzer J. E. Croughan J. Ratcliff K. S. (1981). National Institute of Mental Health diagnostic interview schedule: its history, characteristics, and validity. Arch. Gen. Psychiatry 38, 381–389. doi: 10.1001/archpsyc.1981.01780290015001 6260053

[ref59] Roothman B. Kirsten D. K. Wissing M. P. (2003). Gender differences in aspects of psychological well-being. S. Afr. J. Psychol. 33, 212–218. doi: 10.1177/008124630303300403

[ref60] Russel J. (1980). Three dimensions of emotion. J. Pers. Soc. Psychol. 9, 1161–1178.

[ref61] Salsman J. M. Bingen K. Barr R. D. Freyer D. R. (2019). Understanding, measuring, and addressing the financial impact of cancer on adolescents and young adults. Pediatr. Blood Cancer 66:e27660. doi: 10.1002/pbc.27660, PMID: 30756484 PMC6777708

[ref62] Schwartz R. M. Caramoni G. L. (1989). Cognitive balance and psychopathology: evaluation of an information processing model of positive and negative states of mind. Clin. Psychol. Rev. 9, 271–294. doi: 10.1016/0272-7358(89)90058-5

[ref63] Scorsolini-Comin F. Fontaine A. M. G. V. Koller S. H. Santos M. A. D. (2013). From authentic happiness to well-being: The flourishing of positive psychology. Psicol. Reflex. Crit. 26, 663–670. doi: 10.1590/S0102-79722013000400006

[ref64] Seta J. J. Haire A. Seta C. E. (2008). Averaging and summation: positivity and choice as a function of the number and affective intensity of life events. J. Exp. Soc. Psychol. 44, 173–186. doi: 10.1016/j.jesp.2007.03.003

[ref65] Sfeir E. Geara C. Hallit S. Obeid S. (2020). Alexithymia, aggressive behavior and depression among Lebanese adolescents: A cross-sectional study. Child Adolesc. Psychiatry Ment. Health 14, 1–7. doi: 10.1186/s13034-020-00338-2 32939221 PMC7487493

[ref66] Sharma V. Marin D. B. Koenig H. K. Feder A. Iacoviello B. M. Southwick S. M.. (2017). Religion, spirituality, and mental health of US military veterans: results from the National Health and resilience in veterans study. J. Affect. Disord. 217, 197–204. doi: 10.1016/j.jad.2017.03.071, PMID: 28415007

[ref67] Sharma S. Singh K. (2019). Religion and well-being: the mediating role of positive virtues. J. Relig. Health 58, 119–131. doi: 10.1007/s10943-018-0559-5, PMID: 29353383

[ref68] Srivastava K. (2009). Urbanization and mental health. Ind. Psychiatry J. 18, 75–76. doi: 10.4103/0972-6748.64028, PMID: 21180479 PMC2996208

[ref69] Tiliouine H. (2014). “Happiness in Islam,” in Encyclopedia of Quality of Life and Well-Being Research (Dordrecht: Springer), 2662–2667.

[ref70] Trzebiński J. Cabański M. Czarnecka J. Z. (2020). Reaction to the COVID-19 pandemic: the influence of meaning in life, life satisfaction, and assumptions on world orderliness and positivity. J. Loss Trauma 25, 544–557. doi: 10.1080/15325024.2020.1765098

[ref71] Vandekerckhove J. Matzke D. Wagenmakers E.-J. (2014). Model Comparison and the Principle of Parsimony. eScholarship, University of California.

[ref72] Yaacoub H. Hallit S. Haddad C. Zoghbi M. Dib T. Kazour F. (2019). Association of war and Other Factors with substance use in a Lebanese male sample. J. Drug Issues 49, 106–117. doi: 10.1177/0022042618807765

[ref73] Zweig J. S. (2015). Are women happier than men? Evidence from the Gallup world poll. J. Happiness Stud. 16, 515–541. doi: 10.1007/s10902-014-9521-8

